# Multiscale Length Structural Investigation and Thermoelectric Performance of Double-Filled Sr_0.2_Yb_0.2_Co_4_Sb_12_: An Exceptional Thermal Conductivity Reduction by Filler Segregation to the Grain Boundaries

**DOI:** 10.1021/acsmaterialsau.3c00107

**Published:** 2024-02-16

**Authors:** Federico Serrano-Sanchez, João Elias Rodrigues, Javier Gainza, Catherine Dejoie, Oscar J. Dura, Neven Biskup, Norbert M. Nemes, José Luis Martínez, José Antonio Alonso

**Affiliations:** † Instituto de Ciencia de Materiales de Madrid (ICMM), Consejo Superior de Investigaciones Científicas, c/Sor Juana Inés de la Cruz 3, E-28049 Madrid, Spain; ‡ CELLS−ALBA Synchrotron Light Source, Cerdanyola del Valles, Barcelona E-08290, Spain; § ESRF − European Synchrotron Radiation Facility, 38000 Cedex Grenoble, France; ∥ Departamento de Física Aplicada, Universidad de Castilla-La Mancha, E-13071 Ciudad Real, Spain; ⊥ GFMC, Departamento de Física de Materiales, Universidad Complutense de Madrid, Madrid E-28040 Spain; # Instituto Pluridisciplinar, Universidad Complutense de Madrid, Madrid E-28040 Spain

**Keywords:** thermoelectrics, skutterudites, inhomogeneous filling, phase segregation, thermal conductivity reduction, synchrotron powder diffraction

## Abstract

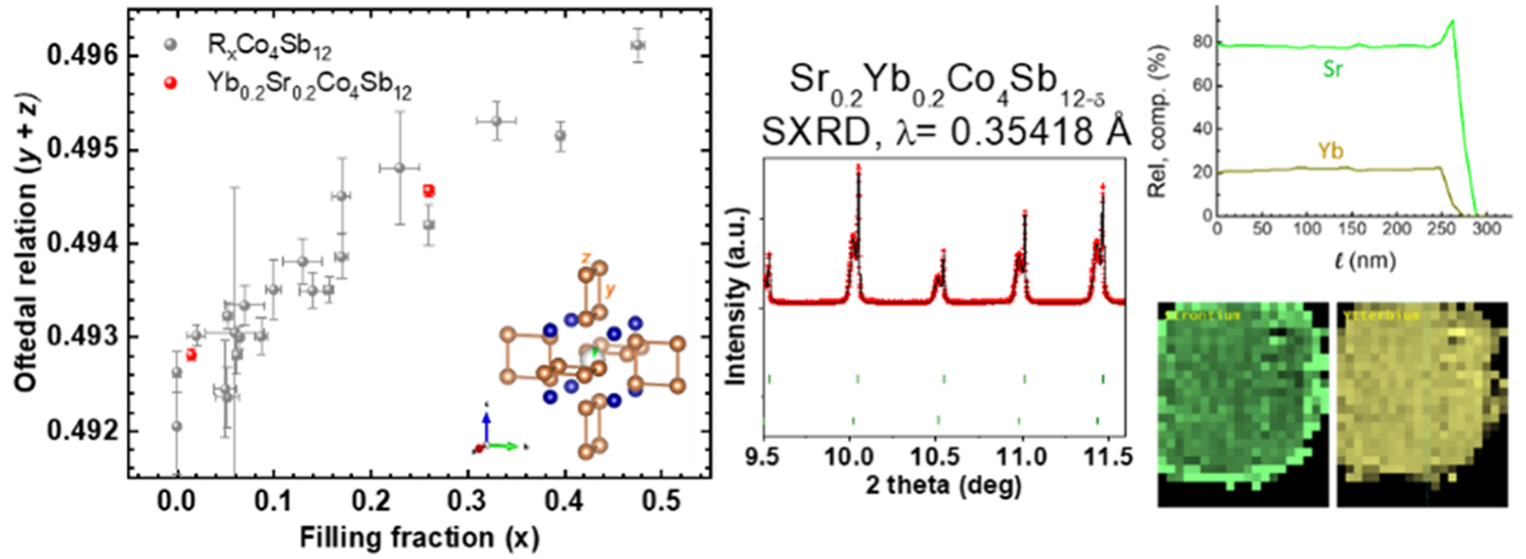

Among thermoelectric materials, skutterudites are the most prominent candidates in the mid-temperature range applications. In the multiple-filled Sr_0.2_Yb_0.2_Co_4_Sb_12_ skutterudite, with Sr and Yb as fillers, we have enhanced the thermoelectric performance of CoSb_3_ through the reduction of lattice thermal conductivity and the optimization of carrier concentration and electrical conductivity. The high-pressure synthesis of the double-filled derivative promotes filling fraction fluctuation. This is observed by high angular resolution synchrotron X-ray diffraction, showing a phase segregation that corresponds to an inhomogeneous distribution of the filler atoms, located at the 2*a* positions of the cubic space group Im3̅. In addition, scanning transmission electron microscopy (STEM) combined with EELS spectroscopy clearly shows a segregation of Sr atoms from the surface of the grains, which is compatible with the synchrotron X-ray powder diffraction results. Mean square displacement parameters analysis results in Einstein temperatures of ∼94 and ∼67 K for Sr and Yb, respectively, and a Debye temperature of ∼250 K. The strong effect on resonant and disorder scattering yields a significantly lower lattice thermal conductivity of 2.5 W m^–1^ K^–1^ at 773 K. Still, good weighed-mobility values were obtained, with high filling fraction of the Yb and Sr elements. This drives a reduced electrical resistivity of 2.1 × 10^–5^ Ω m, which leads to a peak *zT* of 0.26 at 773 K. The analysis and results performed for the synthesized (Sr,Yb)-double filled CoSb_3_, shed light on skutterudites for potential waste-heat recovery applications.

## 1 Introduction

Waste-heat recovery in energy production processes reduces the environmental impact by allowing more efficient and less-polluting sustainable development. The implementation of waste-heat recovery relies in the thermoelectric materials, which enable the direct conversion of thermal gradients into electric energy, and vice versa.^1^ Thermoelectric devices offer significant advantages in terms of their reliability and endurance. Nonetheless, their widespread application has been limited due to low conversion efficiencies. This limitation arises from irreversible losses occurring within thermoelectric materials during the conversion process, which can be evaluated using the thermoelectric figure of merit: *zT* = *T* × *S*^2^σ/κ. Here, *T* represents the absolute temperature, *S* denotes the Seebeck coefficient, σ is the electrical conductivity, and κ stands for the total thermal conductivity, encompassing both lattice and electronic contributions.^2^

In recent years, extensive efforts have been focused on mid- and high-temperature range thermoelectric modules. Among the promising candidates within this temperature range are the skutterudite derivatives of CoSb_3_, which have attractive thermoelectric performance.^3−5^ These derivatives exhibit favorable characteristics, such as good thermal and mechanical stability and low toxicity compared to traditional thermoelectric systems such as PbTe, and they are composed of environmentally friendly elements. The parent compound, CoSb_3_, exhibits high charge carrier mobility and a good Seebeck coefficient owing to its predominantly covalent framework. However, its efficiency is hindered by a high thermal conductivity (∼9 W m^–1^ K^–1^ at 300 K). Consequently, numerous approaches have been employed to decrease κ and thus optimize the thermoelectric behavior of this family of materials. These approaches include nanostructuring, low-dimensionalization, phonon and band engineering through techniques including band convergence and filler-induced phonon resonance scattering.^6^

The crystalline structure of CoSb_3_ belongs to the cubic space group Im3̅, with 32 atoms in the unit cell arranged as Co_8_Sb_24_, where Co occupies 8*c* (1/4,1/4,1/4), Sb occupies 24*g* (0, y, z), and there are empty 2*a* Wyckoff positions. These vacant spaces (also known as voids) can be filled with different elements, materializing the benchmark of the “phonon-glass electron-crystal” concept by Slack,^7^ where the filler acts as a rattler scattering phonons, while the covalent framework as a pathway for charge carriers. Indeed, filling the 2*a* voids in the CoSb_3_ lattice with additional elements has yielded encouraging results, especially when combined with other approaches, such as high-pressure torsion.^4,8,9^ The single-filled derivatives are typically represented by the formula M_*y*_Co_4_Sb_12_, with the filler atoms enclosed in an icosahedral cage formed by tilted [CoSb_6_] octahedra, which are interconnected by Sb–Sb atoms shaping rectangular [Sb_4_] rings. By following this strategy, numerous studies have reported the thermoelectric performance enhancement by using rare-earth,^10−17^ alkali,^18−20^ alkali-earth,^21−24^ and transition-metal elements^25−27^ as fillers. Additionally, multiple-filled skutterudite compounds can be achieved by simultaneously employing different filler elements, leading to enhanced phonon scattering across a wider range of wavelengths.^28,29^ The phonon scattering mechanisms in filled M_*y*_Co_4_Sb_12_ have been extensively discussed, with previous scenarios describing resonant phonon scattering of the rattlers as Einstein-type isolated harmonic oscillators, while further phonon inelastic neutron studies have revealed the coupling of lattice vibrations. Still, analysis of the Einstein and Debye temperatures has typically provided insights into the effect of the filler on the lattice thermal conductivity.^30−35^ Moreover, the filler atoms, being electropositive elements, also electrically dope the material, allowing for charge carrier optimization within the filling fraction limits.

Herein, we focus on filling the CoSb_3_ skutterudite structure with Sr (alkali-earth) and Yb (rare-earth) elements. As individual fillers, they have previously shown significant enhancement of the thermoelectric performance of CoSb_3_ skutterudite. Both single-filled derivatives have demonstrated reduced lattice thermal conductivity and high filling fraction compared to most other filler elements.^11,30,32,33,35−37^ Additionally, high-pressure conditions during the preparation of Sr-CoSb_3_ have resulted in filling fraction fluctuations, leading to the formation of a mixture of two differently filled phases with distinct unit-cell parameters. In this study, we have synthesized the multiple-filled Sr_0.2_Yb_0.2_Co_4_Sb_12_ skutterudite. Synchrotron diffraction experiments reveal the distribution of the fillers in two distinct phases. This scenario is complemented with the results of STEM and EELS regarding the segregation of Sr within the nanometric-size grains. Sb *K*-edge X-ray absorption spectroscopy (XAS) in the extended region (EXAFS) was considered to shed light on the local atomic structure around the antimony absorber, enabling a precise estimation of the lattice dynamics under temperature variation of the covalent environment composed of Sb–Co and Sb–Sb bonds. The thermoelectric transport properties of this compound indicate highly doped samples with high filling fractions, resulting in elevated carrier concentration and electrical conductivity. The Seebeck coefficient of Sr_0.2_Yb_0.2_Co_4_Sb_12_ peaks at much higher temperatures compared to other CoSb_3_ derivatives (723–773 K). Moreover, the lattice thermal conductivity of Sr_0.2_Yb_0.2_Co_4_Sb_12_ is significantly reduced to approximately ∼2–3 W m^–1^ K^–1^ at 773 K, reaching values much lower than those of unfilled CoSb_3_. The charge carrier-weighted mobility in the measured temperature range (30–10 cm^2^ V^–1^ s^–1^) is similar to that of other filled samples.^38^

## 2 Experimental Methods

### 2.1 Synthesis Protocol

The samples were prepared with the nominal composition Sr_0.2_Yb_0.2_Co_4_Sb_12_ using Yb (99.9%, Alfa-Aesar), Co (99%, ROC/RIC), and Sb (99.5%, Alfa-Aesar) in powder form and Sr granules (Alfa-Aesar, 99%). A stoichiometric mixture of 1.0 g was sealed in niobium capsules in inert conditions within a glovebox and then placed in the graphite cylinder heater within a *pyrex* tube. A 3.5 GPa pressure was applied to this setup combined with a temperature of 1073 K in a piston–cylinder press (Rockland Research Co.) for 1 h.^39^ Afterward, it was quenched down to room temperature, and the pressure released. The samples obtained have a cylindrical shape of ∼4 mm diameter and 1–3 mm height, with density above 90% of the crystallographic values. The samples were cut and polished to perform transport measurements and ground to powder for diffraction and microscopic studies.

### 2.2 Structural Characterization

Initial phase characterization was carried out by X-ray diffraction on a Bruker-AXS D8 diffractometer (40 kV, 30 mA), run by *DIFFRACTPLUS* software, in Bragg–Brentano reflection geometry with Cu Kα radiation (λ = 1.5418 Å). Synchrotron X-ray powder diffraction (SXRD) data were collected at room temperature at the ID22 high-angular resolution diffractometer at the ESRF (Grenoble), with a wavelength of 0.35418 Å.^40^ In complement, temperature-dependent SXRD patterns were recorded at the MSPD diffractometer at ALBA Synchrotron Light Source (Barcelona, Spain), at 200, 400, 600, and 800 °C, with λ = 0.45861 Å,^41^ using a hot-air blower system. In both cases, the sample was contained in a 0.5 mm diameter quartz capillary, rotating during data collection to minimize preferred orientation effects. Refinement of diffraction data was carried out with the *Fullprof* program.^42^ The peak shape was analyzed with a pseudo-Voigt function, and the parameters included in the full refinement were scale factors, zero-point error, background coefficients, asymmetry correction factors, lattice parameters, atomic positions, occupancy and isotropic displacement factors of the filler element, and anisotropic displacement parameters for Co and Sb atoms.

### 2.3 X-ray Absorption Spectroscopy

X-ray absorption spectroscopy (XAS) experiments were carried out in transmission mode at Sb *K*-edge at the beamline BL22-CLÆSS^43^ of the CELLS-ALBA (Barcelona, Spain) using an unfocused beam collimated to 3(H) × 1(V) mm^2^. The monochromatic beam was delivered by a pair of Si(311) crystals that defines an energy resolution of Δ*E*/*E* ∼ 2.8 × 10^–5^. The absorption coefficient was estimated from the photon flux detected using ionization chambers placed before and after the sample. The sample preparation for XAS comprises finely ground powders mixed with cellulose, which were pelletized into disks of 13 mm in diameter to achieve an ideal absorption edge jump of ∼1–1.2. For low-temperature acquisitions, a N_2_-flow cryostat was employed, which enabled cooling down to 80 K. The monochromator energy drift was tracked by following the edge energy position of a Sb foil (≥99.95%, Goodfellow).

The EXAFS data were obtained in the *k*-range up to 16 Å^–1^ and alongside the temperature interval 80–340 K taking the following temperatures 80, 100, 140, 180, 220, 260, 301, and 340 K. The EXAFS oscillations χ(*k*) were extracted using *Athena* software,^44^ which enables pre-edge background subtraction, edge jump normalization, and then the extraction of the extended-range oscillations. The Fourier transform (FT) of the *k*-weighted *k*^3^χ(*k*) oscillations was performed using the Hanning type-window at both *k*- and *R*-spaces as Δ*k* = 2–15.2 Å^–1^ and Δ*R* = 1–4 Å, respectively. To fit the EXAFS data using *Artemis* software,^45^ theoretical single scattering paths were calculated from *FEFF* multiple scattering path expansion,^46^ starting from the structural model for CoSb_3_ within the cubic space group Im3̅.^35^ Then, the calculated paths were fitted to the experimental spectra by adjusting the EXAFS signal (*k*-weighted = 1, 2, and 3) to the EXAFS variables, such as average bond distance (*d*_Γ_), bond variance [or Debye–Waller exponent (σ_Γ_^2^)], and average coordination number (*N*_Γ_). The amplitude reduction factor (*S*_0_^2^ ≈ 0.8015) was estimated from EXAFS fitting of the Sb foil spectrum.

### 2.4 Scanning Transmission Electron Microscopy

TEM images with atomic resolution were obtained in a JEOL ARM 200 electron microscope equipped with an aberration corrector. Scanning transmission electron microscopy (STEM) was employed to take annular bright field (ABF) and annular dark field (ADF) images. A Gatan “Quantum” detector was used for electron energy loss spectroscopy (EELS) spectrum images.

### 2.5 Transport Properties

The van der Paw technique was applied to measure the electrical resistivity, while the Seebeck coefficient was determined in an MMR technologies instrument (10^–3^ mbar) under vacuum in the temperature range 300–773 K. the thermal diffusivity (α) was measured in a Linseis LFA 1000 equipment by the laser-flash technique and used to calculate the total thermal conductivity. The equation κ = *αc*_p_*d*, where *c*_p_ is the specific heat, calculated using the Dulong–Petit equation, and *d* is the sample density was employed to determine the thermal conductivity (κ).

## 3 Results

### 3.1 Structural Analysis from SXRD Data

**Figure 1:**
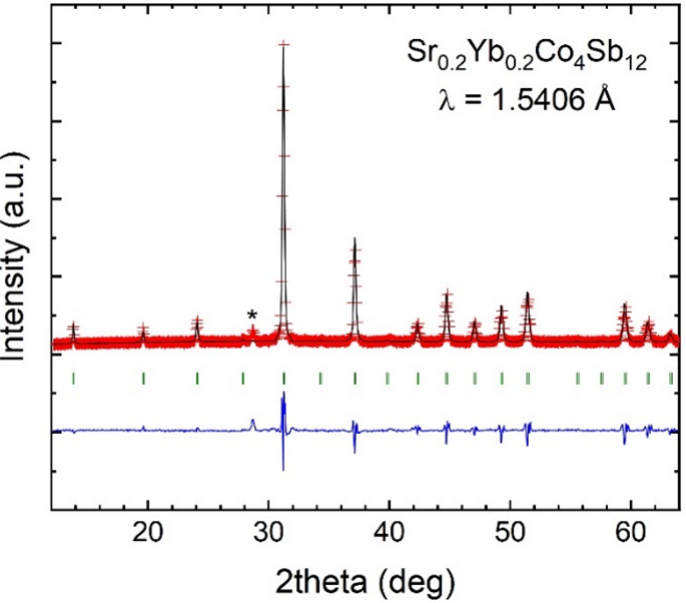
Laboratory XRD pattern of as-grown Sr_0.2_Yb_0.2_Co_4_Sb_12-y_ at room temperature. The star corresponds to the most intense reflection of the Sb impurity. Red crosses are the raw experimental XRD data, black line the calculated profile curve, the blue line represents the difference between experimental and calculated curve, and green bars the Bragg reflections expected for the cubic space group *Im*3̅.

High-pressure synthesis conditions were essential to stabilize the skutterudite-type material in much shorter periods of time (typically 1 h) than in conventional solid-state preparation and also prevent the oxidation of the metal elements (Co, Sb) and in particular the fillers (Sr, Yb). The resulting material, with nominal composition Sr_0.2_Yb_0.2_Co_4_Sb_12-y_ was initially characterized by laboratory X-ray diffraction (XRD). The sample was obtained from the Nb capsules as compact pellets, which were ground into a fine powder for laboratory XRD characterization. The Rietveld refined XRD pattern is shown in Figure 1. The pattern corresponds to a cubic polycrystalline specimen with the characteristic reflections of skutterudite-type phases and a minor amount of unreacted Sb.

**Figure 2:**
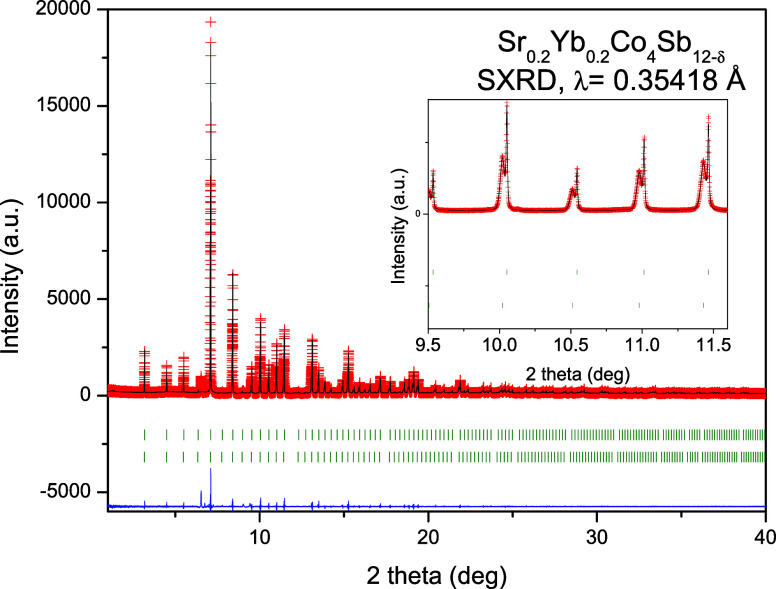
Observed (red crosses), calculated (black solid line), and their difference (blue line) SXRD profiles for Sr_0.2_Yb_0.2_Co_4_Sb_12-δ_ at 295 K, with the peak splitting highlighted in the insets. The two series of Bragg reflections correspond to the two coexisting skutterudite phases.

A SXRD study was important to determine the fine structural features of the synthesized skutterudite (in Figure 2). The crystal structure model was defined in the cubic Im3̅ (No. 204) space group. Co atoms are located at 8*c* (1/4, 1/4, 1/4), Sb atoms occupy the 24*g* (0, y, z) sites, while the filler Sr and Yb atoms occupy the cages at 2*a* (0, 0, 0) Wyckoff positions. It is remarkable that the room temperature SXRD pattern shows a systematic splitting of the diffraction peaks, which is more noticeable at high scattering angles (see the insets of Figure 2). This is not obvious in the laboratory XRD patterns, but it can be appreciated in the SXRD diagram due to its extremely high angular resolution. This splitting is accounted for by admitting the presence of two different skutterudite phases, for which conspicuous differences in unit-cell parameters and filling fraction are found.^47,48^ The formation of these two differently filled phases is expected by using high-pressure synthesis, as observed in other single-filled compositions.^5−7^ In fact, there is some intrinsic pressure inhomogeneity within the niobium capsule, where the regions with direct contact between the grains experience superior mechanical stress; this effect leads to different filling factors and then phase segregation, as observed by high-resolution X-ray diffraction. This multiphase sample nature has been reported to reduce the lattice thermal conductivity to extremely low values as a consequence of additional resonant scattering. A first insight already shows that the first phase (with higher *a* unit-cell parameter) gives rise to broader peaks, where the second phase (with smaller *a* value) exhibits much narrower peaks, corresponding to larger scattering domains (see insets of Figure 2). Table 1 lists the main refined parameters, including occupation factors of the filler atoms independently refined for each phase, unit-cell parameters, atomic positions, phase ratios, bond distances, and reliability factors. The distribution between Sr and Yb in each phase is based on the results of STEM, described below. There is a conspicuous Sb deficiency, as observed in prior specimens synthesized under high-pressure conditions, accounting for the observation of a minor Sb impurity in the X-ray diffractogram. There is a major phase [66.1(5)% in wt.] with a large filling factor of *x* = 0.26, and a correspondingly larger lattice parameter of 9.06779(3) Å, whereas the minor phase [33.9(3)% in wt] exhibits a weak refined filling factor of *x* = 0.03, with a lattice parameter *a* = 9.04012(2) Å. As described below, the transport properties of these compounds and, in particular, the thermal conductivity will be influenced by the segregation in two differently filled phases. Additionally, we performed a temperature-dependent SXRD analysis of all the samples. The phase segregation remains stable up to 873 K. The SXRD patterns at different temperatures are represented in Figure S1 (see the Supporting Information).

**Table 1:** Refined Structural Parameters of Sr_0.2_Yb_0.2_Co_4_Sb_12-δ_ Consisting of Two Skutterudite Phases at Room Temperature Obtained from SXRD Data, Space Group: *Im*3̅

Nominal Composition	Sr_0.2_Yb_0.2_Co_4_Sb_12-δ_	
Refined Compositions	Sr_0.007_Yb_0.007_Co_4_Sb_11.79(3)_	Sr_0.20(1)_Yb_0.05(1)_Co_4_Sb_11.79(3)_
Phase abundance (%)	33.9(3)	66.1(5)
Lattice parameter, *a* (Å)	9.04012(2)	9.06779(3)
Unit-cell volume (Å^3^)	738.793(2)	745.599(4)
β_11_ (Co) (Å^2^)	0.0017(7)	0.0017(7)
β_12_ (Co) (Å^2^)	–0.0001(1)	–0.0001(1)
*y* (Sb)	0.33510(5)	0.33611(5)
*z* (Sb)	0.15770(5)	0.15845(5)
Occ (Sr)	0.015	0.200(4)
Occ (Yb)	0.015	0.050(1)
β_11_ (Sb) (Å^2^)	0.0011(1)	
β_22_ (Sb) (Å^2^)	0.0021(1)	
β_33_ (Sb) (Å^2^)	0.0012(1)	
β_23_ (Sb) (Å^2^)	0.0002(1)	
B (M filler) (Å^2^)	6.0(4)	
*d* (Co–Sb) (Å)	2.5290(3)	2.5373(3)
*d*_1_ (Sb–Sb) (Å)	2.851(1)	2.874(1)
*d*_2_ (Sb–Sb) (Å)	2.981(1)	2.972(1)
*R*_p_ (%)	4.69	
*R*_wp_ (%)	5.71	
*R*_exp_ (%)	3.47	
*R*_Bragg_ (%)	4.03	2.63
χ^2^ (%)	2.71	

### 3.2 X-ray Absorption Spectroscopy

**Figure 3:**
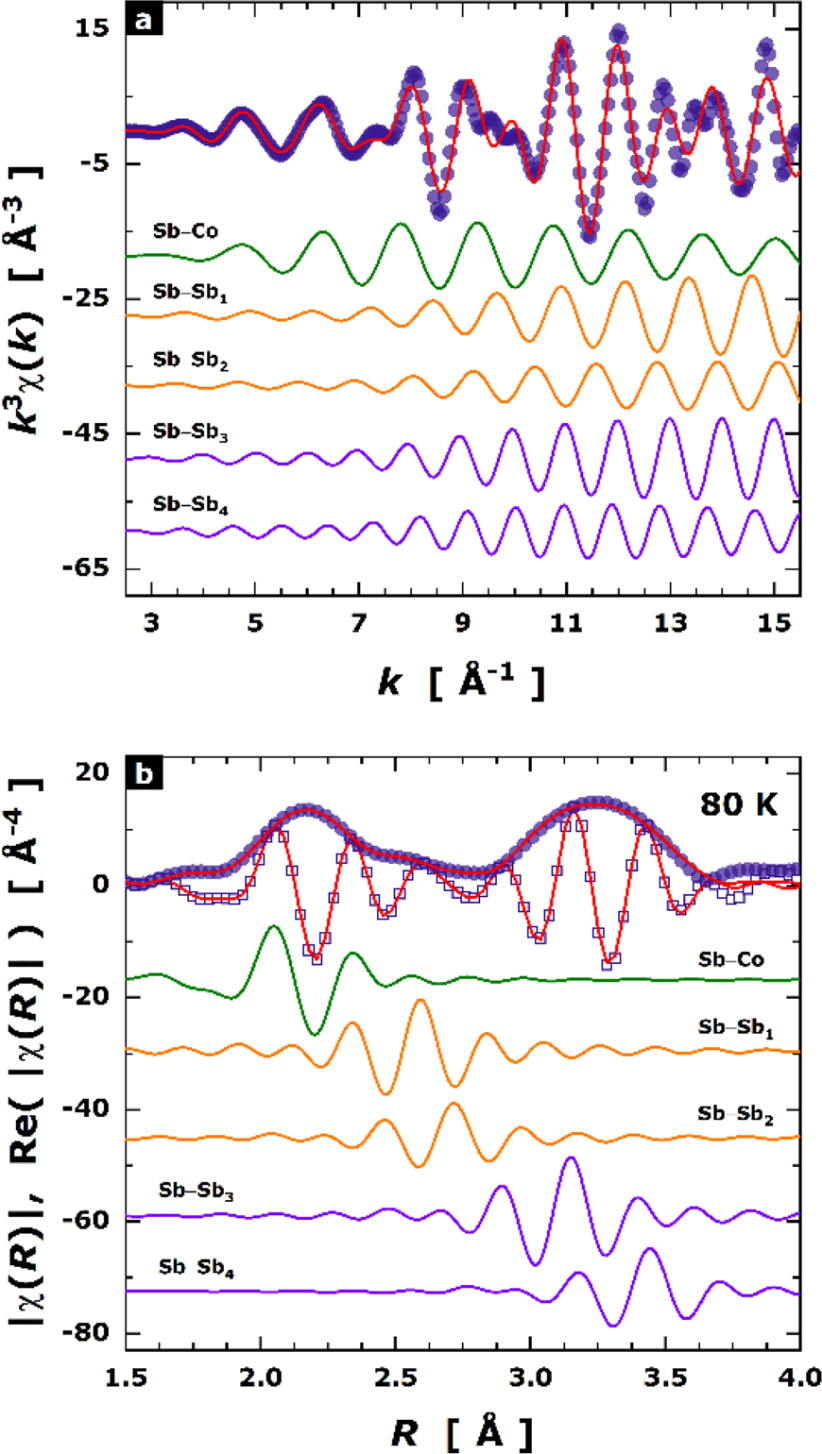
Raw Sb *K*-edge EXAFS oscillation χ(*k*) at 80 K (dark blue open symbols), and the individual fitted scattering paths (green: Sb–Co bond; orange: Sb–Sb_1_ and Sb–Sb_2_ bonds; purple: outer shells Sb–Sb_3_ and Sb–Sb_4_), and the total fitted EXAFS signal (red line). Fourier transform magnitude of *k*^3^χ(*k*) and its real part of the raw data, individual scattering paths, and summed paths [symbols and lines as in (a)].

The EXAFS data provide insight into the local structure of the CoSb_3_-based compound. Initially, we evaluated the local structure of Sr_0.2_Yb_0.2_Co_4_Sb_12_ at the lowest temperature of 80 K. The low temperature condition enables a precise evaluation of the EXAFS oscillations, considering that the Debye–Waller exponent is expected to be reduced as much as possible. In Figure 3, the *k*-weighted raw oscillation *k*^3^χ(*k*) at 80 K is shown in panel (a), while its Fourier-transform is exhibited in panel (b). A first inspection of the Sb *K*-edge EXAFS results of (Sr, Yb) double-filled revealed that its local structure around Sb absorber is very similar to the pristine CoSb_3_ as reported in ref (35). Initial models considering Sb–M (M = Sr, Yb) pair bonds were tested but did not provide reliable fitting parameters. Likely, the backscattering signals generated by Sr and Yb are too small to be detected as a reasonable oscillation. This occurred mainly because Sr/Yb are very diluted in the crystal structure, and then a possible electronic shielding cannot be disregarded. In this sense, the EXAFS modeling was built considering five single scattering (SS) paths for describing the covalent environment composed by [CoSb_6_] and [Sb_4_] units. These paths comprise the Sb–Co bond with theoretical coordination of *N*_1_ = 2, being followed by Sb–Sb bonds (Sb–Sb_1_ and Sb–Sb_2_) composing the [Sb_4_] ring. The outer shells for scattering paths above ∼3 Å (Sb–Sb_3_ and Sb–Sb_4_) were found to play a crucial role to improve the EXAFS fitting to 15.2 Å^–1^ in *k*-space. In Figure 3, the comparison between the experimental and fitted data indeed attested to the reliability of the local structural model. Table 2 lists the best fitted EXAFS variables from the Sb *K*-edge XAS spectrum of Sr_0.2_Yb_0.2_Co_4_Sb_12_ at 80 K. The EXAFS model was then applied to fit the temperature-dependent spectra in the range 80–340 K. The fitting convergence was quite stable with *R*-factors ranging between 0.0158 and 0.0207.

**Table 2:** Local Structural Variables Refined from the EXAFS Fitting^a^

	Sr_0.2_Yb_0.2_Co_4_Sb_12_ EXAFS at 80 K			CoSb_3_ EXAFS at 80 K^35^	
SS path	*d*_Γ_ (Å)	σ_Γ_^2^ (10^–3^ Å^2^)	*N*_Γ_	*d*_Γ_ (Å)	*N*_Γ_
Sb–Co	2.514(8)	2.7(6)	2.6	2.528(7)	2.3
Sb–Sb_1_	2.857(8)	0.3(1)	1.2	2.863(7)	1.2
Sb–Sb_2_	2.981(8)	1.2(1)	1.2	2.993(8)	1.1
Sb–Sb_3_	3.412(8)	1.5(1)	3.0	3.428(8)	3.0
Sb–Sb_4_	3.707(8)	3.4(1)	4.6	3.723(4)	4.3
					
*R*-factor	0.0207				
Δ*k* (Å^–1^)	2–15.2	Δ*R* (Å)	1.5–4		
*N*_idp_	21	*N*_v_, pts	14, 330		

a *d*_Γ_ represents the average path distance, σ_Γ_^2^ is the bond variance (or Debye–Waller exponent), and *N*_Γ_ is the average coordination number. SS stands for single scattering.

**Figure 4:**
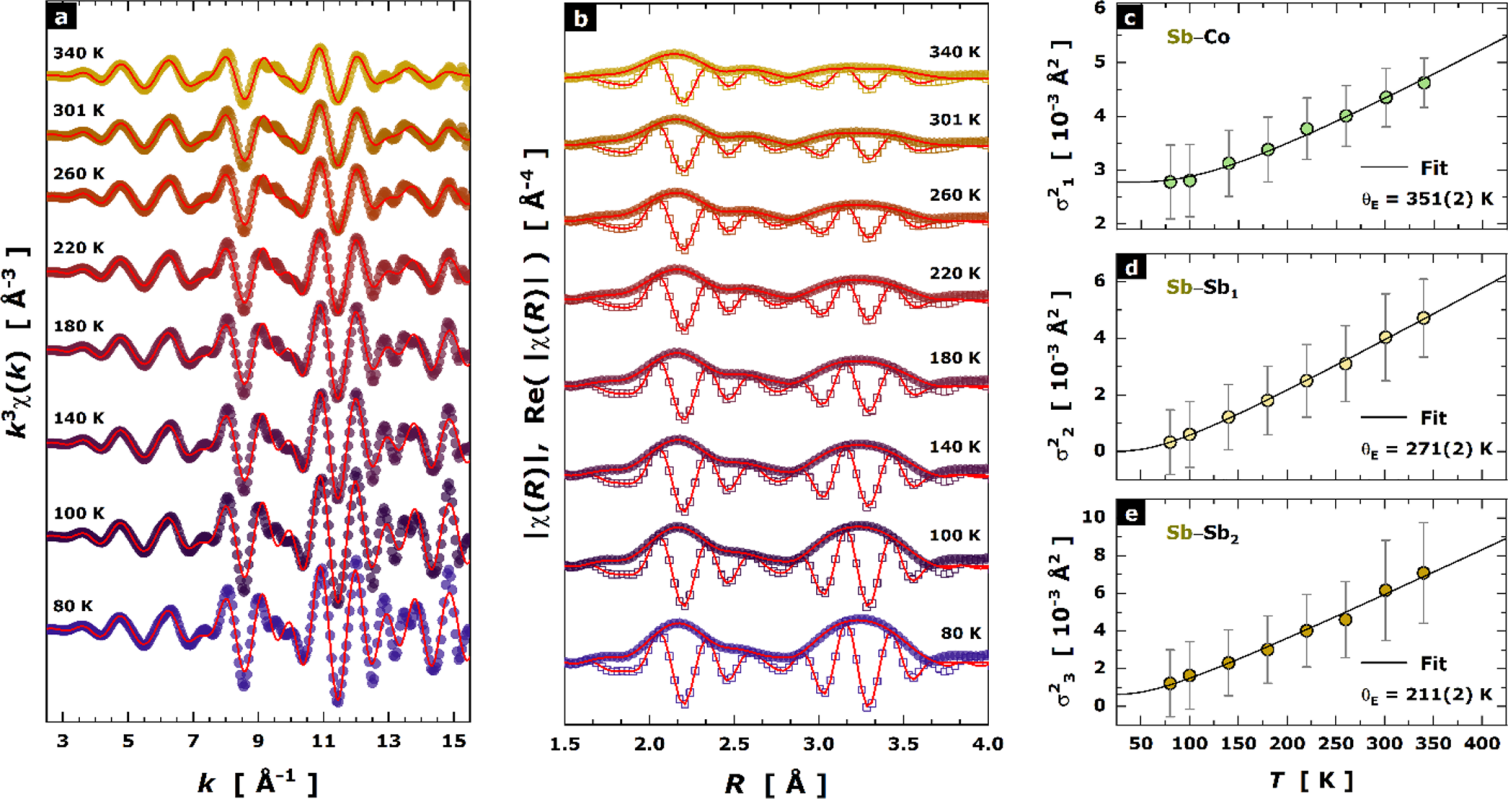
Temperature-dependent EXAFS data at Sb *K*-edge being represented as (a) *k*^3^-weighted oscillations in *k* space, (b) moduli of Fourier transform χ(*R*) and their corresponding real part in *R* space. Open symbols stand for the raw experimental data measured under heating 80–340 K, while the red lines represent the best EXAFS fit. Temperature dependence of the EXAFS fitted Debye–Waller exponent (σ_Γ_^2^) for the paths Co–Sb, Sb–Sb_1_, and Sb–Sb_2_. The black lines denote the best fit of σ_Γ_^2^ values to Einstein’s model.

In Figure 4, the raw and fitted temperature-dependent EXAFS spectra are plotted for temperature variation from 80 up to 340 K. The *k*^3^-weighted raw oscillations *k*^3^χ(*k*) (a), the moduli of χ(*R*)b and its real part (b) as a function of temperature are exhibited. We observed that the previous structural model was successfully applied to all temperature points, and no evidence of structural transformations was found. One may see continuous changes with increasing temperature in the shape/intensity of the moduli, mainly the peak broadening along all the single scattering paths in the *R* range1.7–3.7 Å. At the level of the covalent framework composed by [CoSb_6_] and [Sb_4_] units, the cooling down induced a bond contraction of ∼0.13, 0.35, and 0.26% for the paths Sb–Co, Sb–Sb_1_, and Sb–Sb_2_, respectively. Similarly, no anomalies in the Debye–Waller exponents for these paths were noticed in the range 80–340 K, as demonstrated in Figure 4c–e.

### 3.3 Results from STEM

**Figure 5:**
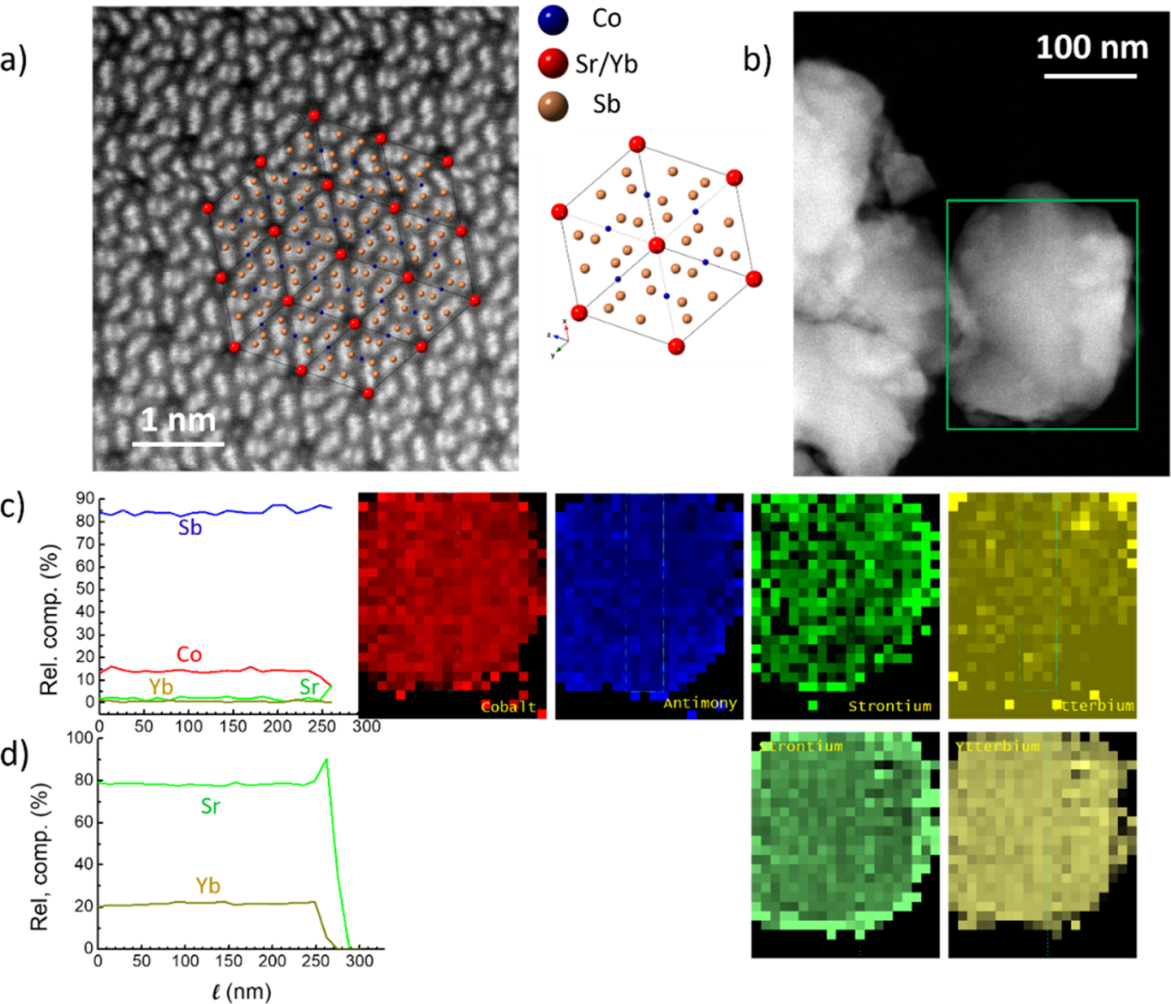
(a) Atomic resolution image of Sr_0.2_Yb_0.2_Co_4_Sb_12_ crystal with Sr/Yb atoms located in the A site of the skutterudite cubic lattice. (b) Crystallite where spectroscopy is taken. (c) EDS elemental maps and their profiles (left) based on Co *K*-edge, Sb, Sr, and Yb *L*-edges. (d) EELS elemental maps of dopants (Sr and Yb) and their profiles (left).

Figure 5 presents the electron microscopy images and spectroscopy of nominal Sr_0.2_Yb_0.2_Co_4_Sb_12_. In panel (a), the skutterudite crystal along the [111̅] zone axis is shown. The filler strontium/ytterbium atoms occupy the A site of the cubic lattice, as indicated in the crystal structure shown in the same panel. In panel (b), the crystallite where spectroscopy was used to reveal the chemical composition is exhibited. In panel (c), the elemental imaging and profiles using energy-dispersive X-ray spectroscopy (EDS) of all four elements are represented. In panel (d), the simultaneous elemental imaging and profile of dopant elements (Sr, Yb) using electron energy loss spectroscopy (EELS) are shown. Elemental maps (based on Co *K*-edge, Sb, Sr, and Yb *L*-edges) are drawn in false colors and their profiles are taken in the vertical direction, as indicated in elemental maps. This particular spectrum-image slightly overestimates the amount of antimony on the expense of cobalt [nominally 73% Sb and 24% Co], but the analysis of other grains exhibits that the concentration is close to the nominal value [78 ± 5% of Sb versus 20 ± 5% of Co]. The two filler atoms are present in small concentrations, but in all cases (all grains examined and in both, EDS and EELS spectra) the amount of strontium is approximately four times larger than the amount of ytterbium [80%–20% shown in profile in panel (d)]. The main feature in Figure 5 of STEM is the inhomogeneity of strontium concentration: strontium tends to build up on the edges of the grain. This can be seen in both EDS and EELS methods, which are complementary in the case of transmission electron microscopy. In these profiles, one may see the strong increase of Sr concentration at the crystallite border. The ytterbium concentration, on the other hand, remains marginal and approximately constant throughout the grain.

These findings from STEM are fully compatible with the results described above from high-resolution SXRD. As previously discussed, the peak splitting is explained by the presence of two types of crystallographic domains. The peaks corresponding to a phase with a higher unit-cell parameter, and therefore with a larger filling fraction, arise from the periphery of the grains, where Sr atoms are preferentially segregated; those peaks are much broader as they stem from relatively narrow diffraction domains. By contrast, the main bulk of the grains, which give rise to much narrower reflections given the extension of the diffraction domains, are characterized by a reduced filling fraction, where a weak but homogeneous distribution of Yb and Sr is observed by EELS (see in Figure 5c and d). This dopant segregation toward grain boundaries region has been observed in other thermoelectric materials, as in half-Heusler compounds, by the solute-drag effect during the preparation process.^49,50^ There, the electrical transport throughout the energy barrier posed by grain boundaries is facilitated and the overall conductivity improved. Besides, the “crust” of the grains, much richer in Sr filler and the derived inhomogeneity that is expected across the solid, can act as a barrier for the phonons, having dramatic consequences in the reduction of the thermal conductivity, as described below.

### 3.4 Transport Properties

**Figure 6:**
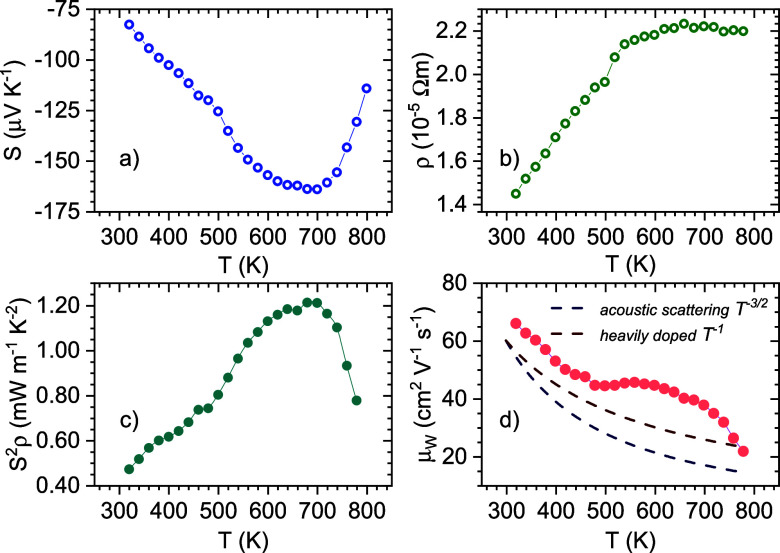
Temperature dependence of the (a) Seebeck coefficient, (b) electrical resistivity, (c) power factor (S^2^ρ), and (d) weighted mobility for the Sr_0.2_Yb_0.2_Co_4_Sb_12_ skutterudite. In panel (d), theoretical weighted mobilities according to acoustic scattering (*T*^–3/2^) and heavily (*T*^–1^) doped models are represented as dotted lines.

The Seebeck coefficient dependent on the temperature is shown in Figure 6a. It follows the trend for a degenerate semiconductor, increasing up to a peak at which bipolar conduction has a significant impact. Remarkably, the maximum absolute values reach a plateau at 650–700 K around ∼165 μV K^–1^, maintaining the peak values at higher temperatures than other filled-skutterudites, indicating a higher carrier concentration through doping by the filler elements and temperature-activated bipolar conduction. These values are below those reported in Sr_*x*_Yb_0.03_Co_4_Sb_12_ (x = 0.21, 0.16), reaching up to −180 μV K^–1^ at 850 K,^51^ while in agreement with those of Sr_0.1_Yb_0.1_Co_4_Sb_12_.^52^ The electrical resistivity evolution with temperature (Figure 6b) exhibits an increase throughout the measurement range, with a small variation from 1.4 × 10^–5^ to 2.1 × 10^–5^ Ω m. Above 500 K, a plateau is observed, an indication of hole thermal activation contributing to electrical transport. These values confirmed the doping of the structure compared to unfilled CoSb_3_ (10^–3^ Ω m), being also near the previously reported for other filled skutterudite prepared by the same method, such as the Sr-, Ce-, K-, and Y-filled CoSb_3_, and similar to those of the Yb-single filled CoSb_3_.^33,53^ Besides, they are slightly below reported values for other filled skutterudites,^25,30,54^ similar to those of Sr_0.1_Yb_0.1_Co_4_Sb_12_,^52^ while slightly larger than those of Sr_*x*_Yb_0.03_Co_4_Sb_12_ (x = 0.21, 0.16).^51^ The overall power factor increases up to 1.20 mW m^–1^ K^–2^ at 773 K, following the same trend as the Seebeck coefficient, with slightly lower values than other filled-CoSb_3_ (1–3 mW m^–1^ K^–2^), as a consequence of the lower absolute Seebeck coefficient values. We can use the weighted mobility (Figure 6d) to compare charge carrier scattering behavior without the need for Hall Effect measurements. In this case, the values are above those of other single filled skutterudites, i.e., near 40–20 cm^2^ V^–1^ s^–1^ and then, based on the low resistivity values, implying that the doping level in this double-filled samples agrees with the low filling fraction described in the R-poor phase by SXRD.^38^ This enhanced weighted mobility can be ascribed to the reduced space-charge effect of grain boundaries scattering due to Sr segregation.^49^ It displays a temperature evolution closer to acoustic phonon scattering than to a heavily doped semiconductor; however, the trend is distorted by the contribution of bipolar activation. This behavior has been observed near the maximum absolute values of the Seebeck coefficient due to the lower doping level compared to higher filling fraction compositions.

**Figure 7:**
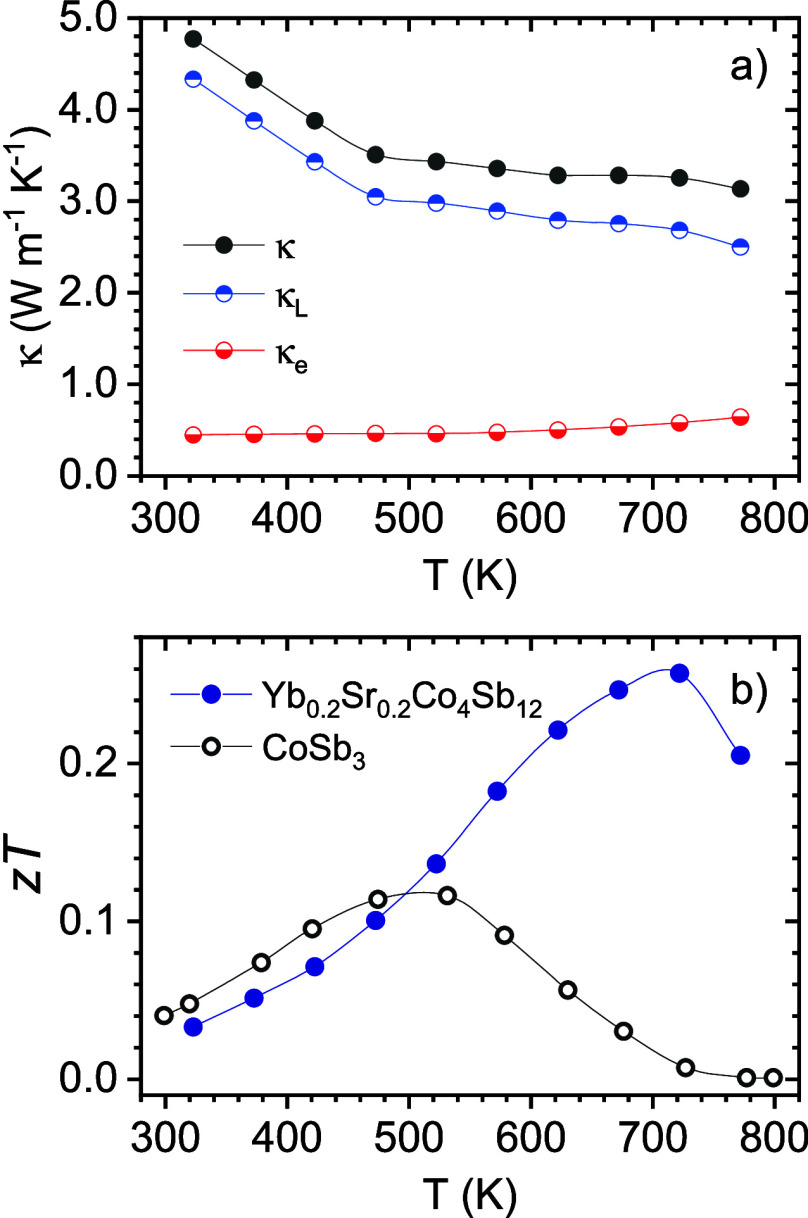
(a) Temperature dependence of the total thermal conductivity with electronic (κ_e_) and lattice (κ_L_) contributions and (b) thermoelectric figure of merit (*zT*) for the Sr_0.2_Yb_0.2_Co_4_Sb_12_ skutterudite.

The total thermal conductivity variation with temperature displays a stark reduction compared to the unfilled CoSb_3_ compound (∼9 W m^–1^ K^–1^) reaching 3 W m^–1^ K^–1^ at 800 K (Figure 7a). These values are similar to those reported previously in (Sr,Yb)-filled Co_4_Sb_12_.^51^ Using the Wiedemann–Franz law, κ_e_ = *L*σ*T*, where *L* is the Lorentz number determined by the approximation proposed by Kim et al.,^55^ σ is the electrical conductivity, and *T* is the absolute temperate, we can determine the electronic and lattice contributions to the thermal conductivity. Here, the electronic contribution is significant and yields a lattice thermal conductivity as low as 2.50 W m^–1^ K^–1^ at 773 K, which is close to that reported in Sr_0.1_Yb_0.1_Co_4_Sb_12_.^52^ This value is better than other filled-CoSb_3_, like (Ba, Ce) double filled skutterudite.^29,53^

The thermoelectric figure of merit (*zT*) reaches 0.26 at 773 K, being close to other single-filled skutterudites, but still lower than other high-filling-fraction compositions (Figure 7b).^53^ This fact comes from the lower doping level at the R-poor phase using simultaneous filling of Sr and Yb compared with the corresponding single-filled compositions, leading to a relatively small enhancement of the Seebeck effective mass by band convergence and resonant phonon scattering. Still, the notable increase of the power factor and reduction of the lattice thermal conductivity improves the thermoelectric efficiency above that of the unfilled and some single-filled CoSb_3_.^56^

## 4 Discussion

### 4.1 Local Bonding

**Figure 8:**
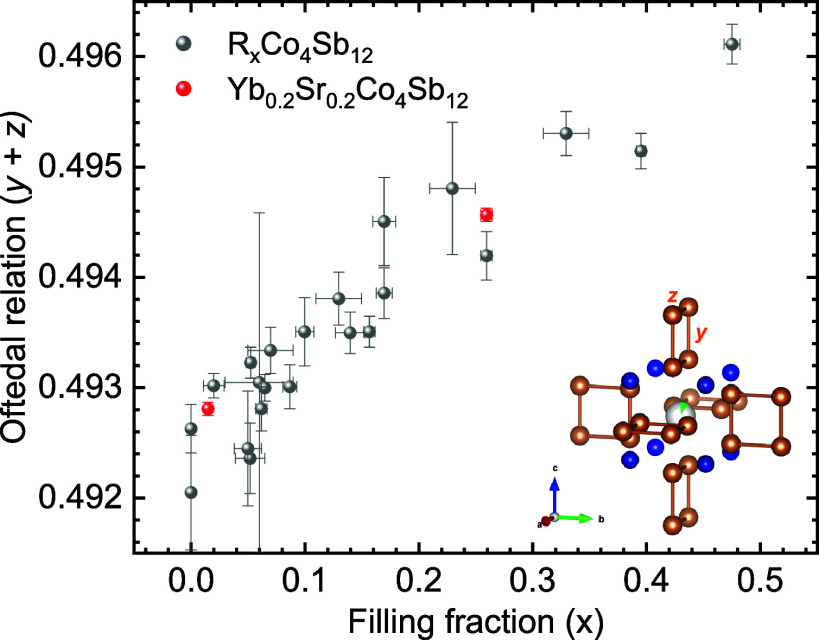
Oftedal (*y* + *z*) Sb positional parameters against filling fraction, with red circles for Sr_0.2_Yb_0.2_Co_4_Sb_12-δ_ filler-rich and filler-poor phases, and gray circles for other single-filled skutterudites described in refs (36), (49), and (54).

There are two main bonding units in the CoSb_3_ skutterudite structure, the tilted [CoSb_6_] octahedron encasing 2*a* voids, and the [Sb_4_] rectangular rings, which link the octahedral grid. In Figure 8, the Oftedal (*y* + *z*) dependence with filling fraction is shown, including data for Sr_0.2_Yb_0.2_Co_4_Sb_12-δ_ and a wide panoply of previously reported single-filled CoSb_3_ compounds.^53^ This plot describes the approximation of [Sb_4_] rings to a square shape (*y* + *z* = 0.5). It is noteworthy that the refinement of independent *y* (Sb) and *z* (Sb) structural parameters for each of the segregated phases is essential to obtain values matching the relation to the filling fraction. Data for refined compositions, such as Sr_0.007_Yb_0.007_Co_4_Sb_11.79(3)_ and Sr_0.20(1)_Yb_0.05(1)_Co_4_Sb_11.79(3)_, agree with the linear dependence of Sb structural parameters with filling fraction, with clear differences compared to Yb and Sr-single filled CoSb_3_. This result further suggests that phase segregation leads to double filled compositions. This evolution of the [Sb_4_] rings upon filling CoSb_3_ toward a more regular configuration has been related to a band convergence scenario of the conduction bands,^57^ optimizing the effective mass for increased thermoelectric efficiency.

### 4.2 Local Thermal Analysis of the Covalent Environment

The EXAFS results enabled a precise evaluation of the lattice dynamics under the temperature variation of the covalent environment composed by the [CoSb_6_] and [Sb_4_] units. For this purpose, we focused on the bond distances of Sb–Co, Sb–Sb_1_, and Sb–Sb_2_. The first one describes the first shell interaction of the photoelectron ejected from the Sb absorber with the Co neighbors, while the last one concerns the edges of the [Sb_4_] ring. To describe the thermal behavior of the bond variance (σ_Γ_^2^) of such paths, we have considered three individual harmonic oscillators as bond vibrations being described by the Einstein’s model,^35,58,59^ as given by
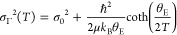
1where θ_E_ represents the Einstein temperature, σ_0_^2^ is the static disorder, μ is the reduced mass of the pair-bond, while *ℏ*, *k*_B_, and *T* maintain their usual physical meaning. From Figure 4c–e, we estimated an Einstein temperature of 351(2) K and a static disorder of ∼1.04 × 10^–3^ Å^2^ for the pair-bond Sb–Co, which is a slightly higher than the Einstein temperature derived for pristine CoSb_3_ [θ_*E*_ = 345 K].^35^ For the pair-bonds forming the [Sb_4_] ring, the Einstein temperatures were estimated as 271(2) and 211(2) K for Sb–Sb_1_ and Sb–Sb_2_, respectively, which are again quite higher than those values for the pristine sample [θ_E_ = 248 and 206 K]. These results demonstrated that the covalent environment became more rigid and, likely, more covalent when the fillers are incorporated into the open cavities of the skutterudite structure. The increase of bonding strength is a consequence of the electron transfer from the filler to the caging structure, which is stabilized under high pressure by Sb vacancies for the unfilled CoSb_3_, and those vacancies are reduced upon filling.^36,47,60,61^

**Figure 9:**
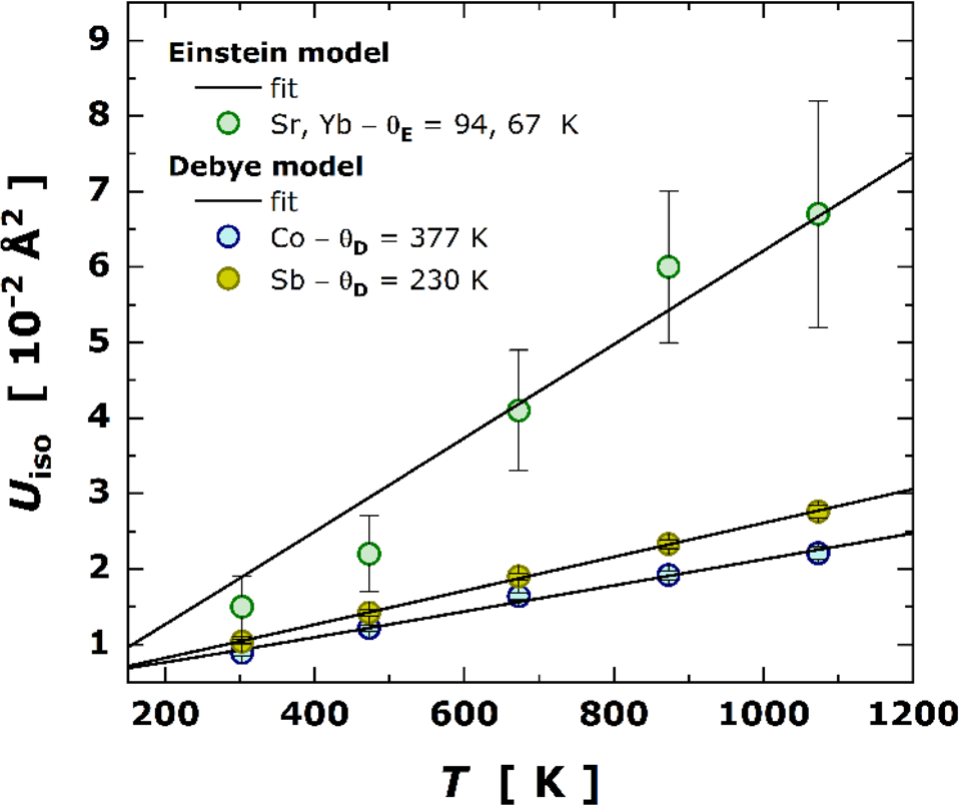
Temperature dependence of *U*_iso_ for Co, Sb, and (Sr and Yb) atoms. Open colored symbols stand for the *U*_iso_ values obtained from the sequential Rietveld refinement. The black solid lines represent the best fits to the experimental data using the Debye/Einstein model.

Similar local structural analysis can be done from the principal thermal-vibration parameters (β_ij_), as converted to the mean-square displacements (MSDs or *U*_iso_, Å^2^), using the Debye’s model as follows:
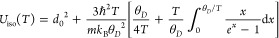
2where θ_D_ stands for Debye temperature, *d*_0_^2^ is the intrinsic disorder (or static displacement at 0 K), and *m* is the atom’s mass. Based on the best fit to model (in Figure 9), we found that Co atom has a Debye temperature θ_D_ of 377 K with an intrinsic disorder of ∼3.8 × 10^–3^ Å^2^, while the Sb atom exhibits a θ_D_ value of 230 K and an intrinsic disorder of ∼3.5 × 10^–3^ Å^2^. Averaging the Debye temperatures by the Co and Sb atomic masses, we can estimate the Debye temperature for the covalent framework of the skutterudite around ∼250 K, which has a reasonable agreement with pristine and other filled skutterudites.^30,36,56^

### 4.3 Local Thermal Analysis of the Filler Elements

At the voids (2*a* positions), Sr and Yb atoms were supposed to act as rattling elements (as explained according to the acoustic phonon scattering model^7^) and then promote the reduction of the thermal conductivity at the atomic level. To evaluate this behavior, we can take advantage again of the Einstein model but now being applied to the mean-square displacements (*U*_iso_), as represented by
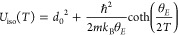
3where *m* represents the atomic mass instead of the reduced mass as in eq 1. In Figure 9, the *U*_iso_ variation for Sr/Yb with temperature is represented together with the best fit to Einstein’s model. Here, the *d*_0_^2^ value was kept equal to zero due to the absence of low temperature points, which could better constrain its precise determination. The Einstein temperatures for Sr and Yb were estimated as 94 and 67 K, respectively. For Yb, the θ_E_ value agrees quite well with those reported for other rare-earth filler elements, such as Gd (∼67 K)^36^ and Eu (∼68 K).^30^ In this way, we may attest that Sr and Yb atoms possess an enormous rattling effect at the skutterudite voids (2*a*), which can explain or at least compose the entire scenario for the exceptionally low thermal conductivity reported in double-filled Sr_0.2_Yb_0.2_Co_4_Sb_12_. In fact, the role of the rattlers (Sr, Yb) occupying the large 2*a* cages of the cubic structure is to contribute to a noteworthy reduction of the thermal conductivity due to the incoherent scattering of phonons derived from their rattling motion.^62−65^

## 5 Conclusions

In this report, we successfully synthesized and investigated the chemicophysical properties of thermoelectric (Sr, Yb)-double filled CoSb_3_ prepared under 3.5 GPa and moderate temperature conditions. Structural characterization by SXRD displayed a filling fraction fluctuation into R-poor *x* = 0.015 and R-rich *x* = 0.25 phases, with a significant rearrangement toward square [Sb_4_] rings of the latter, following the Oftedal relation described for other filled skutterudites. Then, by TEM and EELS analysis, the Yb is found homogeneously distributed throughout the grains, while Sr-rich compositions gather near the grain boundaries area. The study of the mean square displacement parameters yields a Debye temperature of ∼250 K and Einstein temperatures of ∼94 and ∼67 K for Sr and Yb, respectively, and the EXAFS analysis displays an increased Einstein temperature for Sb–Sb and Co–Sb bonds, suggesting a more rigid covalent environment upon filling. The electrical properties display a stark evolution with temperature, marked by bipolar conduction, and improved weighted mobility, possibly due to reduced grain boundary scattering. This indicates a low doping level, associated with the R-poor phase described by the SXRD data. Still, a high weighted mobility and strong reduction of the lattice thermal conductivity (2.5 W m^–1^ K^–1^) yields an improved figure of merit *zT* = 0.26 at 723 K.

## Supporting Information Available

Temperature dependent SXRD patterns for Sr_0.2_Yb_0.2_Co_4_Sb_12_ (PDF)
