# Supplementary material for: Multiscale Length Structural Investigation and Thermoelectric Performance of Double-Filled Sr0.2Yb0.2Co4Sb12: An Exceptional Thermal Conductivity Reduction by Filler Segregation to the Grain Boundaries
Source: ACS Mater Au. 2024 Feb 16;4(3):324–34. doi: 10.1021/acsmaterialsau.3c00107 (PMC11083120; doi:10.1021/acsmaterialsau.3c00107)
Supplement: Supplementary file 1 — mg3c00107_si_001.pdf [file mg3c00107_si_001.pdf]

Supporting Information

Multi-scale length structural investigation and thermoelectric performance of double-filled $Sr_{0.2}Yb_{0.2}Co_4Sb_{12}$: An exceptional thermal conductivity reduction by filler segregation to the grain boundaries

Authors:

Federico Serrano-Sanchez [1], João Elias ~~F. S.~~ Rodrigues [2,3], Javier Gainza [1], Catherine Dejoie[3], Oscar J. Dura [4], Neven Biskup[5,6], Norbert M. Nemes [5], José Luis Martínez [1], and José Antonio Alonso [1]*

Affiliations:

(1) Instituto de Ciencia de Materiales de Madrid (ICMM), Consejo Superior de Investigaciones Científicas, c/ Sor Juana Inés de la Cruz 3, E-28049 Madrid, Spain.

(2) CELLS–ALBA Synchrotron Light Source, Cerdanyola del Valles, Barcelona E-08290, Spain.

(3) ESRF – European Synchrotron Radiation Facility, 38000 Grenoble Cedex, France.

(4) Departamento de Física Aplicada, Universidad de Castilla-La Mancha, E-13071 Ciudad Real, Spain.

(5) GFMC, Departamento de Física de Materiales, Universidad Complutense de Madrid, Madrid, E-28040 Spain.

(6) Instituto Pluridisciplinar, Universidad Complutense de Madrid, Madrid, E-28040 Spain.

Lead contact (*):

ja.alonso@icmm.csic.es

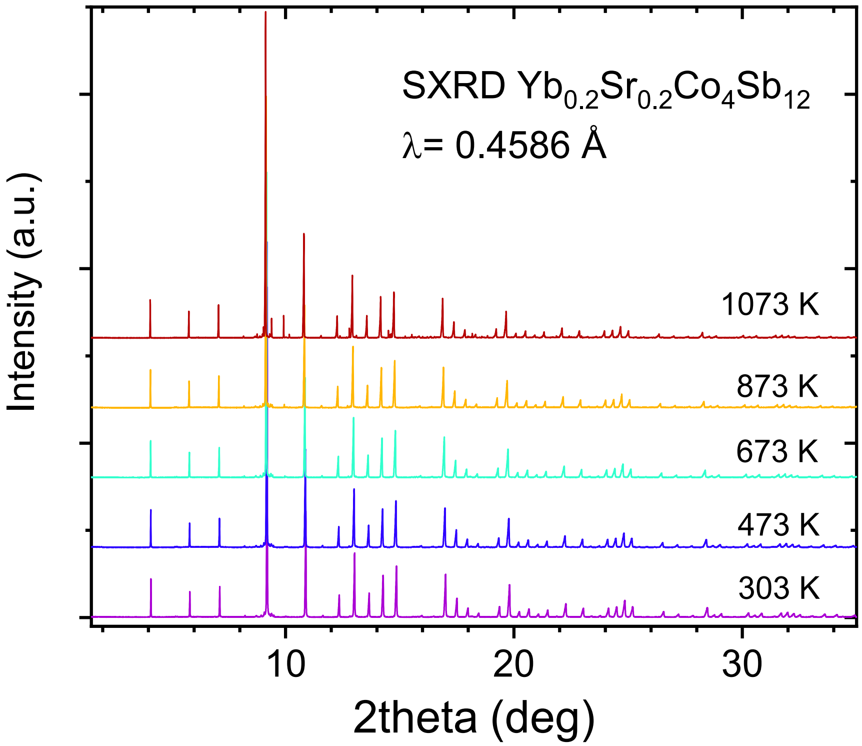

Fig. S1. Temperature dependent SXRD patterns of $Sr_{0.2}Yb_{0.2}Co_4Sb_{12}$ skutterudite at 303, 473, 673, 873, and 1073 K. The patterns were vertically shifted.
